# New Therapeutic Approach for Targeting Hippo Signalling Pathway

**DOI:** 10.1038/s41598-019-41404-w

**Published:** 2019-03-18

**Authors:** Leticia Dominguez-Berrocal, Erica Cirri, Xiguang Zhang, Laura Andrini, Gustavo H. Marin, Sophie Lebel-Binay, Angelita Rebollo

**Affiliations:** 1 PEP Therapy, 45 rue du Cardinal Lemoine, 75005 Paris, France; 2 CIMI Paris, Inserm U1135, 91, bd de l’hôpital, 75013 Paris, France; 3 Facultad de Ciencias Medicas, UNLP-CONICET, 60 and 120, Code, 1900 La Plata, Argentina

## Abstract

Nuclear localization signals are short amino acid sequences that target proteins for nuclear import. In this manuscript, we have generated a chimeric tri-functional peptide composed of a cell penetrating peptide (CPP), a nuclear localization sequence and an interfering peptide blocking the interaction between TEAD and YAP, two transcription factors involved in the Hippo signalling pathway, whose deregulation is related to several types of cancer. We have validated the cell penetration and nuclear localization by flow cytometry and fluorescence microscopy and shown that the new generated peptide displays an apoptotic effect in tumor cell lines thanks to the specific nuclear delivery of the cargo, which targets a protein/protein interaction in the nucleus. In addition, the peptide has an anti-tumoral effect *in vivo* in xenograft models of breast cancer. The chimeric peptide designed in the current study shows encouraging prospects for developing nuclear anti- neoplastic drugs.

## Introduction

One of the main issues to effective drug delivery is the crossing of cell membranes to reach the target. For this purpose, macromolecules require to be specifically delivered in the desired cell compartment. Specific intracellular targeting is advantageous to therapeutic action for several reasons. On one end, the amount of drug necessary to obtain the desired effect may be significantly decreased thanks to its specificity, resulting in maximized therapeutic effect and minimized side effects. On the other end, most importantly, subcellular drug delivery will overcome the main limitation of drug actions, which is multidrug resistance, a major problem in tumor chemotherapy^1^.

Cell penetrating peptides (CPP) have arisen as a new class of shuttles allowing the delivery of molecules across biological membranes. They are used for intracellular delivery of several cargos^2,3^. A CPP^4^ with additional specific targeting features such as the combination with a nuclear localization signal (NLS)^5–7^ or a cargo is a promising targeted approach for therapy. Using such approach, we have generated several cell penetrating and interfering peptides blocking the association between proteins involved in tumoral transformation^8,9^.

Transport of molecules across the nuclear envelope occurs through the nuclear pore complex (NPC)^10–12^. While ions and small molecules can cross by passive diffusion, larger molecules require binding to nuclear transport protein factors called nucleoporins to mediate their translocation across the NPC. Nucleoporins facilitating transport into the nucleus are known as importins^13,14^, which recognize and bind to nuclear localisation signals on the cargo. The cargo in complex with importins can diffuse through the nuclear pore and is then released into the nucleus. NLS are mainly classified in monopartite and bipartite, although other authors establish up to six categories of NLS^15^. Monopartite NLSs are exemplified by the SV40 large T antigen (^126^PKKKRKV1^32^)^16^. Interestingly, mutation of the lysine in second position completely abolishes nuclear import, suggesting the importance of this residue. Bipartite NLSs are exemplified by the nucleoplasmin NLS^16–18^. They contain two groups of basic residues separated by a linker consisting of 10-13 non-conserved amino acids (^155^KRPAATK-KAGQAKKKK^169^). The consensus sequence for classical monopartite NLS is K-K/R-X-K/R, whereas that for the bipartite signal is (K/R)(K/R)X_10–13_(K/R)_3/5_, where X is a non-conserved amino acid and (K/R)_3/5_ indicates three lysine or arginine amino acids out of five consecutive residues^13^.

The Hippo signalling pathway is a major controller of cell proliferation and apoptosis. It is composed of a core of kinases leading to the inactivating phosphorylation of the co-transcriptional activator YAP, (Yes Associated Protein). When the pathway is inactive, YAP is dephosphorylated and translocates to the nucleus where it associates to the TEAD family of DNA binding protein^19,20^. This complex transactivates a large set of target genes involved in cell proliferation and survival. In association to the kinases, the Hippo pathway contains several proteins with regulatory functions. Among them, the Merlin protein, coded by the NF2 gene has been shown to be a key regulator. Loss of Merlin expression leads to constitutive YAP nuclear localization. Indeed, NF2 gene is a tumor suppressor and its inactivation in human triggers the development of intracranial cancers such as schwannomas and meningiomas. A significant percentage of patients affected of liver, breast, lung pancreas and ovarian cancer present an overexpression of YAP^21^. Several evidences suggest that TEAD-YAP complex can be targeted for cancer therapy or to modulate proliferation^22^. YAP transcriptional activity requires its binding to TEAD proteins. Therefore, one attractive strategy for the targeting of YAP consists of preventing its interaction with TEAD using interfering peptides. Using the PEP scan approach, we have recently patented bi-functional peptides consisting of a penetrating sequence (CPP), associated to an interfering peptide blocking the association between TEAD and YAP, two proteins of the Hippo signalling pathway, and *vice versa*. In the present study, we have engineered a peptide combining a NLS with a CPP in order to generate a nuclei-addressed CPP. To validate the potential use of these CPPs with specific nuclear localization, we associated a cargo to apply this technology for specific nuclear drug delivery. The candidate cargo was TEAD or YAP derived peptides since the target protein/protein interaction TEAD/YAP is located in the nucleus. In this manuscript we validated *in vitro* and *in vivo* the new generated tri-functional peptides and demonstrated that the association of a NLS to a CPP enhances the nuclear localization and, as a consequence, the apoptotic and anti-tumoral effect of the associated cargo.

## Results

### Identification of the binding sequence of TEAD to YAP and *vice versa*

**Figure 1:**
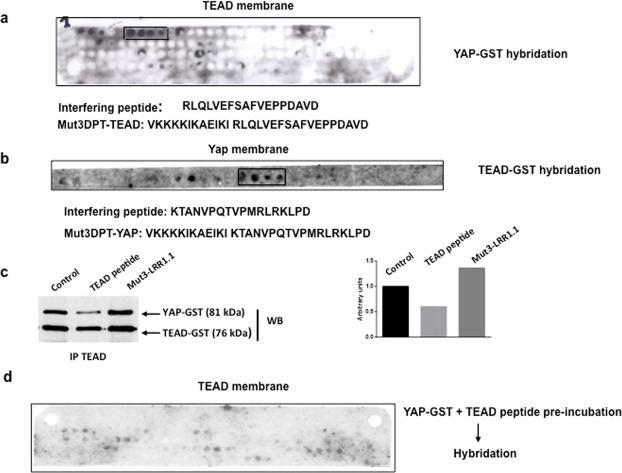
Determination of the binding site of TEAD to YAP and *vice versa*. (**a**) Overlapping dodecapeptides with two amino acid shift covering the whole human TEAD proteins were bound to a solid support. The membrane was incubated sequentially with YAP-GST protein, and anti-GST antibody, followed by a peroxydase-labeled secondary antibody. The membrane was revealed with ECL system. The sequence corresponding to the identified spots is shown. (**b**) Overlapping dodecapeptides with two amino acids shift covering the YAP loop described as binding area to TEAD were synthesized and bound to a solid support. The membrane was incubated with TEAD-GST protein, followed by anti-GST antibody and a secondary peroxidase-conjugated antibody. The membrane was revealed using the ECL system. The sequence corresponding to the identified spots is shown. (**c**) Competition of TEAD/YAP interaction was done *in vitro* using purified proteins and a concentration or 250 μM of TEAD or irrelevant peptide PfMut3 DPT-LRR1.1. Competition was done for 1 h at room temperature. Immunoprecipitates were washed and immunoblotted with anti-YAP antibody and anti-TEAD antibody, as internal control. Densitometry of the protein bands and ratio calculation is also shown. (**d**) Competition of TEAD/Yap was also analysed upon membrane hybridation. Yap-GST protein was pre-incubated with TEAD peptide for 1 h at room temperature. The membrane was incubated with the mix Yap-GST protein/TEAD peptide. After washing steps, the membrane was incubated with anti Yap antibody followed by a peroxiydase-labelled secondary antibody. The membrane was revealed using the ECL system.

To identify segments of the TEAD sequence able to bind to YAP, the whole sequence of TEAD was synthesized as series of overlapping dodecapeptides that were bound to a nitrocellulose membrane, which was incubated with purified GST-tagged YAP. Complex formation was revealed with a labelled anti-GST antibody. Using the PEP scan approach, we identified one binding sequence, of four dodecapeptides corresponding to the binding site of TEAD to YAP. The identified sequence of 18 amino acids, (RLQLVEFSAFVEPPDAVD), corresponds to the amino acids 226 to 244 of TEAD (Fig. 1a).

Similarly, to identify the segment of the YAP sequence able to bind to TEAD, a series of dodecapeptides covering the loop of YAP protein described as involved in binding to TEAD were synthesized and bound to a solid support and incubated with purified GST-tagged TEAD. The sequence identified was KTANVPQTVPMRLRKLPD (Fig. 1b). To demonstrate that the specific target of TEAD peptide is the complex TEAD/YAP, we analyzed whether the peptide was able to target the *in vitro* interaction between both proteins. The interaction was competed with the peptide TEAD (Fig. 1c). YAP was detected in control immunoprecipitates or in immunoprecipitates competed with an irrelevant peptide (Mut3DPT-LRR1.1) while the complex was inhibited when using TEAD peptide for the competition. Figure 1c also shows the ratio YAP/TEAD upon densitometry of the protein bands. TEAD was used as internal control showing similar intensity in all conditions.

To confirm the specificity of the peptide for the interaction TEAD/Yap, we made a pre-incubation of Yap-GST protein with the peptide TEAD before hybridation of the PEP scan TEAD membrane. The pre-incubation abolish the association of Yap protein to TEAD peptides in the nitrocellulose membrane (Fig. 1d and supplementary information).

Taken together, these results suggest that TEAD peptide specifically targets the interaction between TEAD and YAP.

We then synthesized two chimeric peptides composed of a cell penetrating peptide, Mut3DPT-Sh1^8^, associated to the binding site of TEAD to YAP and *vice versa*, as identified above, generating the bi-functional peptides Mut3DPT-TEAD and Mut3DPT-YAP (Fig. 1a,b, Table 1). Given that the TEAD/YAP interaction occurs in the nucleus, we decided to generate new nuclei-addressed cell penetrating peptides, as vehicles conferring specific nuclear localization. We have selected a new cell penetrating peptide (CPP) with optimized internalization and stability properties, Mut7 DPT^4^, and associated it to a bipartite NLS, generating two new cell penetrating peptides, NLS18 and NLS23 (Table 1).

**Table 1:** List of peptides used in this study.

**DPTMut7**	CPP			
	KKKKKWKKWKKK			
**Mut3DPT-TEAD**	CPP	Interfering Peptide		
	VKKKKIKAEIKI	RLQLVEFSAFVEPPDAVD		
**Mut3DPT-YAP**	VKKKKIKAEIKI	KTANVPQTVPMRLRKPD		
**NLS18**	NLS	CPP	NLS	Interfering Peptide
	RKR	KKKKKWKKW	PKKKKLD	
**NLS18-TEAD**	RKR	KKKKKWKKW	PKKKKLD	RLQLVEFSAFVEPPDAVD
**NLS18-YAP**	RKR	KKKKKWKKW	PKKKKLD	KTANVPQTVPMRLRKPD
**NLS23**	NLS	CPP	NLS	Interfering Peptide
	RKR	KKKKKWKKWKKK	PKKKKLD	
**NLS23-TEAD**	RKR	KKKKKWKKWKKK	PKKKKLD	RLQLVEFSAFVEPPDAVD
**NLS23-YAP**	RKR	KKKKKWKKWKKK	PKKKKLD	KTANVPQTVPMRLRKPD

### Quantification of peptides internalization

**Figure 2:**
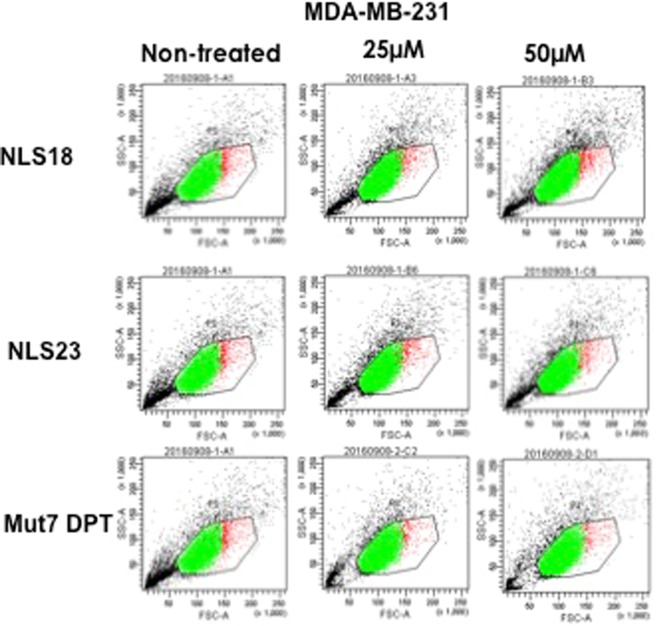
Analysis of toxicity of NLS18 and NLS23 peptides. (**a**) MDA-MB-231 cells (6 × 10^4^ cells/ml) were incubated for 4 h with two different concentrations of NLS18 and NLS23 peptide. Toxicity was analyzed by flow cytometry. Mut7 DPT treated, as well as non-treated cells, were used as control.

**Figure 3:**
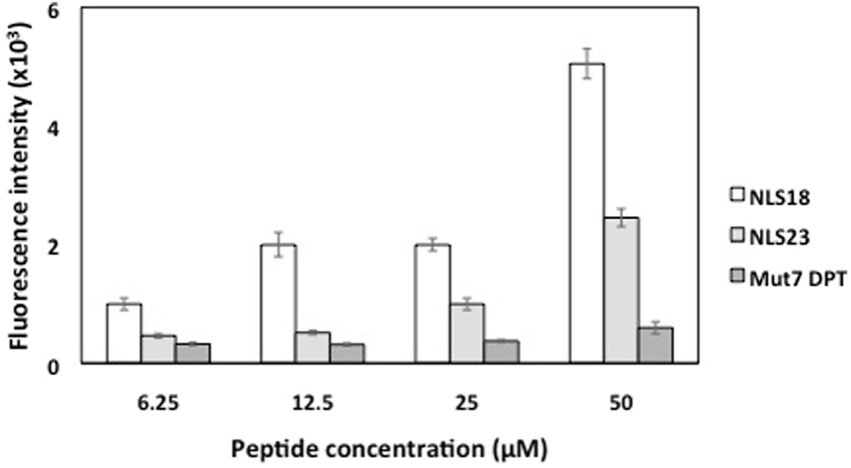
Concentration-dependent internalization of FITC-labelled NLS18 and NLS23. MDA-MB231 cells (5 × 10^4^ cells/ml) were incubated 4 h with different concentrations of FITC-labeled NLS18 and NLS23. The mean fluorescence intensity was detected by flow cytometry and compared to cells treated with the CPP Mut7 DPT control peptide. Standard deviation is shown.

We first analyzed the cell toxicity of NLS18, NLS23 as well as of Mut7 DPT as control (CPP without NLS sequence). The FITC-labeled NLS18 andNLS23 peptides were incubated with the MDA-MB231 cell line at 25 and 50 μM and the toxicity was analysed by flow cytometry (FACS Canto II) upon 4 h of incubation. As shown in Fig. 2, none of the peptides showed toxicity, whatever the concentration used. Similar result was obtained when using the CPP control Mut7 DPT. We then evaluated if FITC-labelled NLS18 and NLS23 could be internalized into cells. Mut7 DPT-FITC was used as a control. MDA-MB231 cells were incubated with FITC-labelled peptides at different concentrations for 4 h and their nuclear penetration was analysed by flow cytometry. Figure 3 shows the internalization of the NLS18 and NLS23 compared to Mut7 DPT control peptide in terms of fluorescence intensity. Both peptides showed higher internalization than the control peptide. Between the two new generated peptides, NLS18 showed better internalization than NLS23 (Fig. 3). Taken together, the new generated CPP-NLS (NLS18 and NLS23) showed an improved internalization profile compared to the control Mut7 DPT without cell toxicity.

### Intracellular localization of NLS18 and NLS23

**Figure 4:**
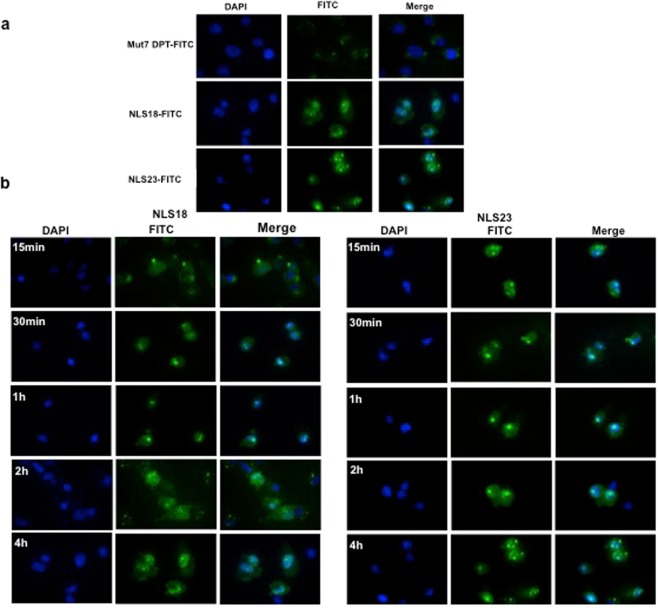
Intracellular localization of FITC-labelled NLS18 and NLS23. (**a**) MDA-MB-231 cells (3 × 10^4^ cells/ml) were grown in Labtek chamber slides cover slips and incubated 4 h with 30 μM of FITC-labelled peptides. Cells were washed 3 times with PBS, fixed with 4% paraformaldeyde (PFA) and analyzed by fluorescence microscopy. (**b**) Time-dependent nuclear localization of FITC-labelled NLS18 and NLS23. MDA-MB-231 cells (3 × 10^4^ cells/ml) were grown as above and incubated with 30 μM of peptide for different periods of time. Cells were washed 3 times with PBS, fixed with 4% PFA and analyzed by fluorescence microscopy.

To confirm the flow cytometry internalization and to visualize the intracellular localization of NLS18 and NLS23, we incubated MDA-MB231 cells with FITC-labelled peptides and peptide intracellular localization was analyzed by fluorescence microscopy following 4 h of incubation at a concentration of 30 μM (Fig. 4a). We observed a punctuate nuclear staining for NLS18 and NLS23 with low cytoplasmic staining. Cells treated with the peptides and non-fixed with PFA confirmed the result obtained using fixed cells (data not shown). This result discards a possible biased subcellular localization because of the hypothetical increase in cell permeability caused by fixation. The control Mut7 DPT-FITC peptide did not show nuclear staining and was mostly distributed in the cytoplasm.

We further analyzed the effect of the incubation time on the penetration at a fixed peptide concentration of 30 μM (Fig. 4b). Among them, NLS23 was the most rapidly internalized peptide, being in the nucleus upon 15 min of incubation with the cells while NLS18 was detected in the nucleus only after 30 min of incubation (Fig. 4b). At 1 h and 2 h of incubation there was not difference compared to 30 min and upon 4 h, most of the peptides were in the nucleus. Taken together, NLS18 and NLS23 showed a better cellular penetration than the control, Mut7 DPT-FITC, and presented a specific nuclear localization.

### Effect of a cargo addition on the properties of NLS18 and NLS23

**Figure 5:**
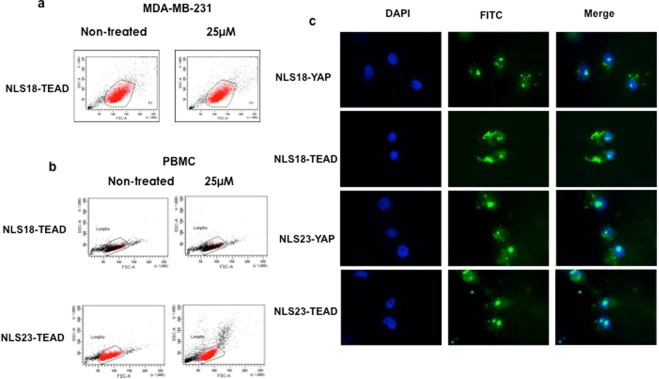
Cytotoxicity and intracellular localization of FITC-labelled NLS18 and NLS23 associated to TEAD/YAP interfering peptides. (**a**) MDA-MB-231 cells were incubated 4 h with NLS18-TEAD peptide. Toxicity was analysed by flow cytometry. Non-treated cells were used as control. (**b**) Peripheral blood mononuclear cells (PBMC) were incubated 4 h with NLS18-TEAD or NLS23-TEAD peptides. Toxicity was analyzed as above. Non-treated cells were used as control. (**c**) MDA-MB231 cells (3 × 10^4^ cells/ml) were incubated in Labtek chamber slides cover slips for 3 h with 15 μΜ of the FITC-labelled NLS18 and NLS23 associated to a cargo, the interfering peptide blocking the interaction between TEAD and YAP proteins and *vice versa*. Cells were washed 3 times with PBS, fixed with 4% PFA and analyzed by fluorescence microscopy.

We analyzed the toxicity of the peptides NLS18-TEAD and NLS23-TEAD. The FITC-labelled peptides were incubated with breast cancer cell line MDA-MB-231 and the peripheral blood mononuclear cells (PBMC, primary cells) isolated from healthy donors. The toxicity was analysed by FACS and as shown in Fig. 5a,b, no toxicity of the peptides was observed, independently of the cell used at the concentration of 25 μM.

We further analysed the impact of the addition of the TEAD or YAP interfering peptides to the CPP-NLS (NLS18 and NLS23) on their nuclear localization. The new generated tri-functional peptides (NLS18-TEAD, NLS18-YAP, NLS23-TEAD, and NLS23-YAP) were labelled with FITC and incubated with MDA-MB231 cells for 3 h at 15μM final concentration. Figure 5 shows that the association of TEAD or YAP to NLS18 or NLS23 does not affect their nuclear localization of the tri-functional peptides, showing similar staining than that observed on Fig. 4a,b. Taken together these results show that the addition of the cargo TEAD or YAP does not modify the original properties (cell penetration, no toxicity and specific nuclear localization) of the NLS18 and NLS23 peptides.

### Apoptotic effect of the tri-functional peptides NLS18-TEAD and NLS23-TEAD

**Figure 6:**
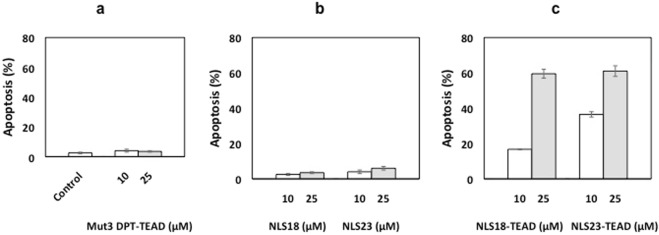
Detection of apoptosis induced by the NLS18-TEAD and NLS23-TEAD. MDA-MB231 cells (5 × 10^4^ cells/ml) were cultured in the presence of different concentrations of NLS18 and NLS23 associated to the interfering peptide blocking the interaction TEAD/YAP for 24 h. Apoptosis was estimated by Annexin V-FITC staining. Cells treated with or without NLS18, NLS23 without a cargo or Mut3DPT-TEAD (without nuclear localization signal) were used as controls. Standard deviation is shown.

We further analysed whether the weak apoptotic effect of the original designed Mut3DPT-TEAD peptide was increased with the newly generated tri-functional peptides having an enhanced shuttle and a NLS. MDA-MB231 cells were cultured for 24 h with 10 and 25 μM of NLS-18, NLS-23, NLS18-TEAD, NLS23-TEAD and apoptosis determined by Annexin V-FITC staining (Fig. 6). The reference peptide Mut3DPT-TEAD induced a very low apoptotic effect. In this experiment, untreated cells were used as control (Fig. 6a). Cells treated with NLS18 and NLS23 were tested to discard the possibility of an apoptotic effect induced by the use of CPPs. As shown on Fig. 6, NLS18 and NL23 peptides did not induce apoptosis in MDA-MB231 cells.

The association of the TEAD interfering peptide to the NLS18 or NLS23 peptides strongly increased the apoptotic effect up to 45 and 63% at a dose of 25 μM, compared to the control peptide (Mut3DPT-TEAD) or the peptides without cargo NLS18 and NLS23. The use of NLS18 and NLS23 to deliver TEAD-derived peptide specifically in the nucleus allows TEAD to exert its biological function at the compartment where the target protein is detected.

### Resistance of the CPP to proteases degradation

**Figure 7:**
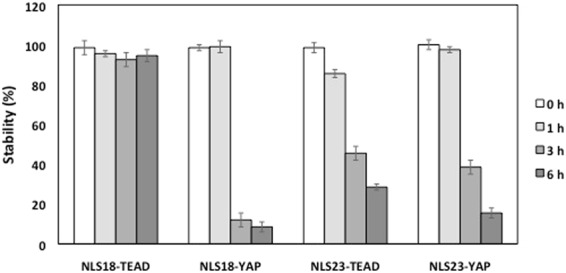
Stability of the new generated peptides in human serum. Peptides were incubated at 37 °C in human serum for different periods of time and their integrity (percentage of intact peptide) was analyzed by mass spectrometry (MS). Every measurement was performed in triplicate. Standard deviation is shown.

We analyzed the stability to proteases degradation in human serum of the CPP Mut7 DPT associated to the NLS and the cargo TEAD or YAP upon incubation at 37 °C for different times. NLS18-TEAD, NLS18-YAP, NLS23-TEAD and NLS23-YAP were incubated with human serum and the resistance to proteases degradation analyzed by mass spectrometry (MS). We detected degradation upon 6 h of incubation in serum of the peptides NLS18-YAP and NLS23-YAP (Fig. 7). On the contrary, NLS18 TEAD showed a constant stability among the time kinetics analyzed. Moderated degradation was detected in NLS23-TEAD upon 6 h of incubation with human serum (Fig. 7). These results suggest that the stability of the interfering peptide is differentially affected by the association of the cargo YAP, while the cargo TEAD does not modify the stability (NLS18-TEAD) or moderately (NLS23-TEAD).

### Anti-tumoral effect of NLS18-TEAD on breast cancer xenograft models

**Figure 8:**
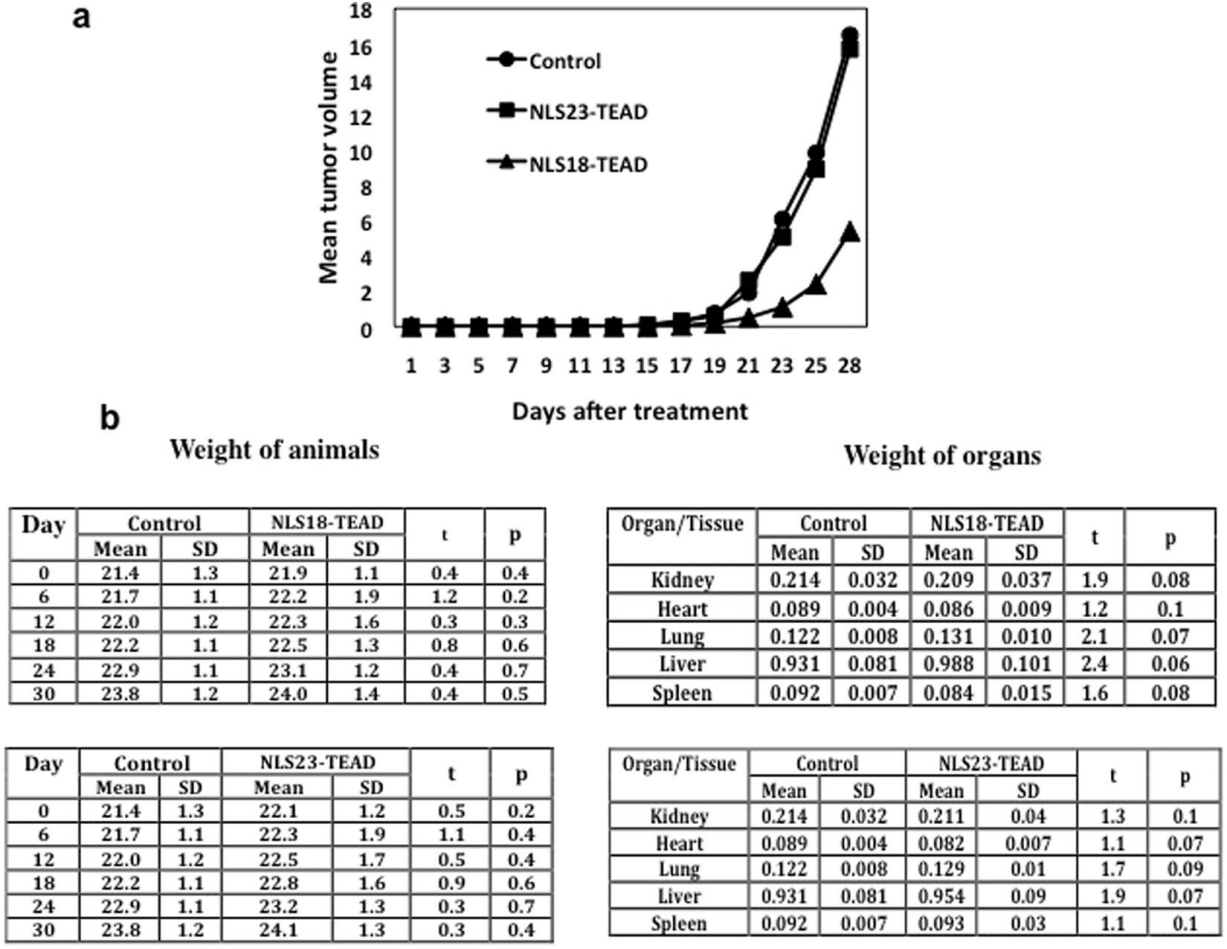
NLS18-TEAD has anti-tumoral effect on breast cancer xenograft models. (**a**) C3H/S-strain mice subcutaneously inoculated with 4 × 10^5^ TN60-UNLP cells were intraperitoneally treated with 5 mg/kg of NLS18-TEAD or NLS23-TEAD 5 days per week for 4 weeks. Control mice received NaCl. Tumor growth was monitored each other day. The average tumor volume of each group (8 mice per group) is shown (p < 0.018). (**b**) The weight of the animals and the organs upon 30 days of treatment with the peptides NLS18-TEAD or NLS23-TEAD is shown. Control non-treated mice are also shown. p and t statistic values are shown.

The therapeutic effect of NLS18-TEAD and NLS23-TEAD was evaluated in a xenograft model of breast caner generated using the cell line TN60-UNLP. The treatment was initiated 5 days after the injection of the cells in C3H/S-strain mice. As shown in Fig. 8, treatment of mice with NLS18-TEAD at 5 mg/kg induced a significant reduction of the tumor size of around 67% compared to control mice treated with saline solution. This tumor reduction was statistically significant (p < 0.018). Treatment of the mice with 5 mg/kg of NLS23-TEAD did not show any reduction in the tumor size compared to control mice (Fig. 8a). The absence of anti-tumoral effect of NLS23-TEAD could be due to the low stability of the peptide in serum (Fig. 7). Figure 8b shows the weight of control and treated animals, as well as the weight of organs at the end of treatment, demonstrating a good tolerance profile of the peptide administered to mice during 4 weeks.

Overall, these results show that we have generated a nucleus-targeted peptide, NLS18-TEAD, with a strong anti tumoral effect *in vivo* due to the specific nuclear targeting of a protein/protein interaction in the nucleus. This peptide can be a promising tool for targeting the Hippo signalling pathway in cancer.

## Discussion

Cell penetrating peptides are capable to transport different types of cargos in the cells. However, although some of them are able to penetrate in the nucleus, most of them do not have a preferential nuclear localization, as nuclear membrane constitutes a barrier for these peptides. The specific drug delivery in the nucleus would result in drug being delivered in the environment of the drug target, maximizing therapeutic effect and minimizing side effects.

In a previous study, we have validated an efficient synthetic Cell Penetrating Peptide (CPP) generated combining lysine and tryptophan residues to increase penetration while keeping the resistance to proteases degradation^4^. In this manuscript, the whole or partial sequence of this CPP, termed Mut7 DPT, was associated to a bipartite nuclear localization signal in order to provide the capacity to cross the nuclear membrane and gain specific nuclear localization. The size of the new generated CPP-Nuclear Localization Signal (NLS) peptides varies between 19 and 22 residues, a size that normally escapes the presentation by MHC-II, which has a preference for longer sequences^23^. According to our results, the use of bipartite NLS constitutes a useful strategy to provide nuclear localization to a CPP, but a compatible CPP is required to fit the demands of importin proteins to be recognized and translocated to the nucleus.

Flow cytometry and fluorescence microscopy experiments revealed that the new generated CPP-NLS, NLS18 and NLS23, have enhanced penetration and specific nuclear localization, compared to the CPP Mut7 DPT alone, and showed no toxicity, suggesting that they could be used at therapeutic level for nuclear cargo delivery. The therapeutic potential of this construction to deliver a cargo specifically in the nucleus was analyzed using an interfering peptide blocking the interaction between TEAD and YAP as a cargo^19^. TEAD and YAP are two proteins involved in the Hippo signalling pathway, which is a major modulator of tissue homeostasis, cell proliferation, tumoral transformation and apoptosis^24,25^. The core of the pathway consists of a series of kinases leading to the phosphorylation of two transcriptional co-activators, YAP and TAZ. When upstream kinases are inactive, YAP and TAZ are not phosphorylated and translocate to the nucleus, binding to TEAD^20^. Deregulation of the Hippo pathway is involved in a broad variety of tumors, including breast cancer^26–34^, therefore, its pharmacological targeting represents an interesting approach for treatment of cancers that harbor functional alterations of this pathway^35^. As an example, one of the small molecules used to target this signalling pathway is Verteporfin, which associates to YAP and inhibits binding to TEAD^36^. The currently available agents acting on the Hippo pathway are not completely satisfactory, as they either have partial impact or low specificity, causing a variety of undesirable medical effects. Therefore, the development of new compounds acting on this Hippo pathway is urgently needed.

The Hippo signalling pathway is a significant controller of tissue homeostasis, cell growth organ dimension and tumoral transformation^24,25^. The most important effectors of this pathway are YAP/TAZ, that act as oncogenes in several human malignancies such as ovarian, non-small cell lung cancer, uveal melanoma, gastric, colorectal, endometrial, breast, and hepatocellular cancer^26,37–45^. Another important regulator in this pathway is the transcription factor TEAD^27^.

Nuclear localization signals are small peptide sequences that promote the nuclear internalization of proteins by binding to their receptors called importins^28–32^. Importins distinguish two types of NLS: monopartite and bipartite. There are two classes of monopartite NLS, one with at least 4 consecutive basic amino acids and another with only three basic amino acids. The consensus for conventional monopartite NLS is K-K/R-X-K/R whereas that for bipartite is (K/R)(K/R)X_10–12_ (K/R)_3/5_, whereas X is an amino acid and (K/R)_3/5_ corresponds to 3 Lys or Arg residues out of 5 successive residues^16,18,32^. Nuclear import can be disturbed by modifications within NLS motif itself. If the import receptor does not identify the NLS-cargo, the cargo remains in the cytoplasm, which could be adverse if its nuclear function is decisive for the cell^33,34^.

We have identified interfering peptides blocking the interaction between the Hippo signalling pathway proteins TEAD/YAP. The addition of a CPP-NLS to these peptides addresses them to the nucleus, where the interaction occurs. Our results suggest that the original properties of CPP-NLS are not modified by the addition of a cargo and allow the transport of TEAD and YAP interfering peptides to the nucleus^22^. An added value of the interfering peptide is its potential protein/protein interaction targeting specificity according to structural information available. If we observe all available TEAD-2 crystal structures, they show the same folding. In these structures, we can observe that the residues comprising our TEAD interfering peptide are part of a β-sheet exposed in the surface of the protein, accessible for eventual interaction with other proteins, which validate our binding site identification in the context of a protein/protein interaction between TEAD and YAP. Furthermore, the sequence corresponding to our peptide targeting TEAD/YAP-2 interaction is only partially conserved among TEAD 1–4 proteins^22^, being TEAD-2 the most different among them, with conserved residues in the central area of the peptide, partially conserved at the N-terminal and non-conserved at the C-terminal. This partial specificity could provide a selected blockage of the interaction between YAP and TEAD-2, but not with other TEADs, increasing its therapeutic interest.

In summary, the tri-functional peptides NLS-18TEAD and NLS23-TEAD constitute a promising anticancer drug candidate that harbour several desired properties such as solubility, rapid cell penetration, no toxicity, specific nuclear localization and apoptotic and anti-tumoral effect in breast cancer xenograft models without toxic effect since we did not detected clinical symptoms in the injected animals, compared to control non-treated animals. No modifications neither in weight of the animals, nor in the weight of organs at the end of the treatment were detected. All these properties would allow an improvement of the pharmacokinetic properties of these peptides (dose reduction, reduced toxicity, lower cost) and avoid eventual off-target effects. Taken together, NLS18-TEAD is an interesting drug candidate to be developed as an anti-cancer therapeutic agent to modulate Hippo signalling pathway.

## Methods

### Synthesis of peptides

Peptides were synthesized in an automated multiple peptide synthesizer with solid phase procedure and Fmoc chemistry by Synpeptide (Shanghai, China) or Smart Biosciences (Saint Egreve, France). The purity and composition of the peptides were validated by reverse phase HPLC and by mass spectrometry (MS) as previously described^4^. The peptides were also synthesized whit a fluorochrome (FITC) in C-terminal.

### TEAD/YAP binding assay on membrane-bound peptides including TEAD or YAP sequences

Overlapping dodecapeptides covering the whole human TEAD-2 protein or the loop of YAP described as binding area to TEAD were prepared by automated spot synthesis into an amino-derivatized cellulose membrane as previously described^46^. The membranes were blocked, incubated with purified TEAD or YAP GST-tagged proteins and, after various washing steps, incubated with anti-GST antibody followed by the PO-conjugated secondary Ab (Dako, Les Ulis, France), following the protocol previously described^4,8^. Protein interaction spots were detected using the ECL system.

### Protein/protein interaction competition

Competition experiments were carried on by initially incubating 500 nM recombinant human ^4^TEAD-GST and YAP-GST purified proteins (Abnova Germany) for 6 h at 4 °C to allow complex formation. The complex was then incubated for 1 h at RT with or without 250 µM TEAD interfering peptide, as well as with 250 µM of the irrelevant peptide PfMut3-LRR1.1 (VKKKKIKAEIKIIENLQNCKKLRLLELGYNKIRM, cell penetrating and interfering peptide blocking the interaction between two proteins of *Plasmodium falciparum* parasite, PP1 and LRR1) to verify the specificity of the competition. The amount of complex present was estimated by western blot with specific anti-TEAD and anti-YAP antibodies (Cell Signaling) following immunoprecipitation with an anti-TEAD antibody. Images were obtained with an ImageQuant LAS 4000 camera system (GE Healthcare) and analyzed with the software ImageQuant TL.

### Cell culture

Human breast cancer cell line MDA-MB231 was cultured in DMEM medium (Gibco, Fisher scientific) complemented with 10% of inactivated fetal bovine serum (FBS) as previously described^4^.

### Quantification of cellular internalization

Cell line MDA-MB-231 was seeded in 24 well plates (5 × 10^4^ cells/well). Cells were cultured with several concentrations of FITC-labelled peptides for different periods of time. Cells were treated with trypsin for 5 min to remove non-internalized surface associated peptide and to detach cells and then, centrifuged, rinsed and resuspended in 200 μl of PBS (Gibco, Fisher Scientific). FITC mean fluorescence intensity of internalized peptide was estimated by flow cytometry (FACS Canto II, BD Biosciences, New Jersey, USA) as previously described^4^. Non-treated cells were utilized as control.

### Detection of apoptosis by Annexin-V staining

The apoptosis induction of the NLS-shuttles associated to the cargo (TEAD or YAP interfering peptides) was analyzed by flow cytometry of cells labelled with annexin V-FITC staining (e Biosciences, Fisher Scientific). Human breast cancer MDA-MB231 was treated with different concentrations of peptide for 24 h. After that, cells were harvested, rinsed and treated following to the manufacturer’s protocol. The level of apoptosis was detected by flow cytometry (FACS Canto II, BD Biosciences, New Jersey, USA). Peptide Mut3 DPT-TEAD and non-treated cells were used as control.

### Visualization of internalized peptides

To determine the intracellular localization of FITC-labeled peptides, MDA-MB231 cells were seeded in an 8-well Labtek chamber slide (Thermo Fischer, Massachusetts, USA) at a concentration of 3 × 10^4^ cells/ml. Cells were treated with 30 μM FITC-labelled peptides for different times and then fixed with 4% of paraformaldehyde for 10 min at room temperature. Samples were rinsed twice with Phosphate Buffered Saline (PBS) and mounted in mounting solution containing DAPI, following our described protocol^4,8^. The experiment was repeated with non-fixed samples to discard a fixation biased localization. Images were captured with a fluorescence microscope (Olympus, Tokio, Japan) using 63x magnification objective.

### Analysis of peptide integrity in human serum

Peptides were incubated at 37 °C in 250 μl of human serum for several times. Samples were collected and peptide degradation stopped by freezing. Peptides were extracted from samples using the Proteo Miner Protein Enrichment System (Bio-Rad, California, USA). Percentage of intact peptide was estimated by mass spectrometry (MS) using MALDI-TOFF (Brucker Autoflex II, Massachusetts, USA) following their protocols. Measurements were performed in triplicates. MS data were analyzed using appropriated software (Clinprot tools, FLEx analysis, Brucker, Massachusetts, USA) as previously described^8,9^.

### Mice

Adult male C3H/S-strain mice of 60 days of age raised in the bioterium of Cytology, Histology and Embryology “A” of the Faculty of Medical Sciences of the National University of La Plata were included in the experiment. Mice were weighed at the beginning of the experiment and each 48 h until the end of the experiment at day 28.

### *In vivo* activity

After a time of synchronization, the C3H/S- breast adenocarcinoma cell line TN60-UNLP was grafted into the subcutaneous tissue of each animal’s flank. Morphologically it is categorized as a solid neoplasm, encapsulated with cells of medium to large size, vesicular nuclei with prominent nucleoli, scarce basophilic cytoplasm and abundant figures of mitosis and apoptotic forms. The dose of the tumor injected subcutaneously on the flank of each mouse was 4 × 10^5^ cells.

A total of 24 graft-bearing mice were split into 3 groups: control injected with saline buffer and two groups injected either with NLS18-TEAD or NLS23-TEAD. The treated groups were injected intraperitoneal with the peptide (5 mg/kg) every day from day 5 during four weeks and then a follow-up with observation, measurement and registration was performed every two days until the fulfillment of the 4 weeks from the start of the trial.

The tumor volume was calculated following formula:

$$(4{\rm{\pi }}/3)\times {(\mathrm{width}/2)}^{{\rm{2}}}\times (\mathrm{length}/2).$$

tumor growth was measured and recorded every two days in all groups.

#### Ethical considerations

Conditions concerning animal management totally respected the policy and conditions of the Guide for the Care and Use of Laboratory Animal Research of the National Research Council. All the experimental protocols and methods were approved by the ethical committee of the University de La Plata, Facultad de Ciencias Medicas, in accordance with the National Guide of the Ministry of Health.

### Statistical analysis

The *in vitro* data are the mean ± SD of three different experiments. For *in vivo* data, 10 animals were included per group and statistical comparisons among groups were performed using Wilcoxon Test.

## Supplementary information

Supplementary information
