# Supplementary material for: New Therapeutic Approach for Targeting Hippo Signalling Pathway
Source: Sci Rep. 2019 Mar 18;9:4771. doi: 10.1038/s41598-019-41404-w (PMC6423280; doi:10.1038/s41598-019-41404-w)

**NEW THERAPEUTIC APPROACH FOR TARGETING HIPPO SIGNALLING PATHWAY**

**Leticia Dominguez-Berrocal^1^, Erica Cirri^1^, Xiguang Zhang^2^, Laura Andrini^3^, Gustavo H. Marin^3^, Sophie Lebel-Binay^1^ and Angelita Rebollo^2^***


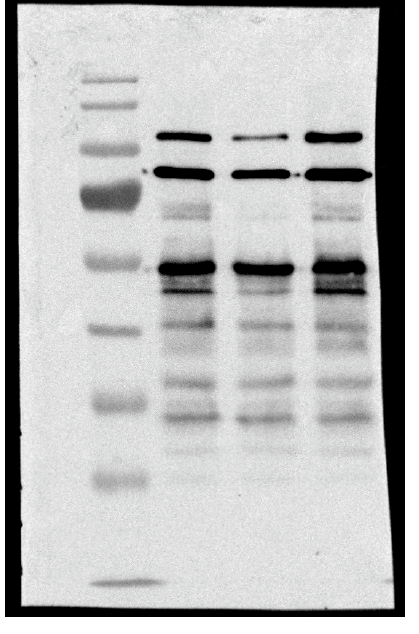

Supplement: Supplementary file 1 — Supplementary information [file 41598_2019_41404_MOESM1_ESM.docx]
